# The role of probiotics in modulating the gut microbiota as a potential inhibitor of diabetic kidney disease progression

**DOI:** 10.1590/2175-8239-JBN-2025-0143en

**Published:** 2026-01-12

**Authors:** Vitoria Cecilia Souza Costa, Monique Moreira Pinheiro, Giulia Triolo Cabreira, Isabella Bacci Bustelli, Julia Ferreira Santos, Sara Ventura, Luciana Soares Costa Santos, Maria de Fatima Fernandes Vattimo, Eloiza de Oliveira Silva

**Affiliations:** 1 Universidade de São Paulo, Escola de Enfermagem, São Paulo, SP, Brazil.; 2 Universidade de São Paulo, Faculdade de Medicina, Instituto do Coração, Laboratório de Biologia Vascular, São Paulo, SP, Brazil.; 3 Faculdade de Ciências Médicas da Santa Casa de São Paulo, Departamento Enfermagem e Fisiologia, São Paulo, SP, Brazil.

**Keywords:** Diabetic kidney disease, Type 2 diabetes mellitus, Probiotics, Oxidative stress

## Abstract

**Introduction:**

Gut dysbiosis is commonly observed in patients with diabetic kidney disease (DKD) and may contribute to its pathogenesis. Among microbial metabolites, butyrate plays a key role in regulating antioxidant proteins in type 2 diabetes mellitus (T2DM). Based on this, we hypothesized that the administering probiotics to diabetic rats modulates redox status and thereby attenuates renal disease progression.

**Methods:**

An in vivo study was performed using 15 male Wistar rats (8 weeks old, 250–300 g) randomized into three groups (n = 5/group): Control (vehicles: 0.9% saline and 0.1 M citrate, pH 4.2, i.p., on day 1), T2DM (nicotinamide 100 mg/kg, i.p., followed by streptozotocin 60 mg/kg, i.p., in 0.1 M citrate buffer, pH 4.2), and T2DM + Prob (T2DM protocol plus a multistrain probiotic—*Bifidobacterium longum*, *Bifidobacterium bifidum*, and *Lactobacillus rhamnosus*—10^10^ CFU/mL by gavage for 6 weeks). The parameters evaluated were: serum creatinine, inulin clearance, microalbuminuria, urinary and lipid peroxides, glutathione, and nuclear factor erythroid 2–related factor 2 (Nrf2).

**Results:**

Probiotic treatment significantly increased Nrf2 expression and glutathione levels, reduced urinary and lipid peroxidation, and—beyond attenuating oxidative stress—improved renal function, with lower serum creatinine and microalbuminuria and higher inulin clearance.

**Conclusion:**

These findings indicate that probiotics prevented DKD progression, likely by modulating oxidative stress via the gut microbiota. These results suggest that probiotics may serve as renoprotective agents, potentially reducing DKD morbidity in T2DM.

## Introduction

Type 2 diabetes mellitus (T2DM) is defined as a constellation of metabolic abnormalities marked by persistently elevated blood glucose levels resulting from reduced pancreatic insulin secretion and/ or decreased insulin sensitivity. It accounts for approximately 91% of the global diabetes burden and is the leading cause of chronic kidney disease (CKD) and end-stage kidney disease (ESKD). T2DM is associated with major cardiovascular risk factors, including hypertension, dyslipidemia, obesity, insulin resistance, and impaired glucose tolerance^1,2^.

Although complications of T2DM have a multifactorial origin, sustained hyperglycemia, by increasing inflammatory cytokines and oxidative stress and thereby promoting long-term injury, appears to be a principal driver of tissue damage in the diabetic kidney. This leads to alarming chronic complications such as retinopathy, neuropathy, cardiovascular disease, and diabetic kidney disease (DKD)^3,4^.

Clinically, DKD is defined by persistent albuminuria ≥30 mg/g (creatinine) and/or a glomerular filtration rate (eGFR) < 60 mL/min/1.73 m^2^ for at least 3 months, irrespective of etiology. It is a chronic complication, resulting in progressive damage and impairment of renal function^5,6^.

DKD is a significant concern worldwide, affecting millions of people with T2DM. Approximately 50% of patients with DKD present microalbuminuria that progresses to macroalbuminuria, one of the most important microvascular complications of DKD, with half of all ESKD cases related to the complication of DM^5^.

Thus, DKD is one of the main causes for initiating renal replacement therapy and is associated with increased morbidity and mortality. Therefore, it is urgent to explore effective, low-cost treatments to reduce morbidity and mortality in patients with DKD^3,4^.

Beyond traditional treatment, such as glycemic control and pharmacotherapy, new therapeutic approaches are being explored. Among these, the promising role of probiotics in the management of DKD stands out. Recent studies have shown that probiotics can play an important role in the prevention and treatment of DKD through different mechanisms, such as modulation of the gut microbiota^7,8^.

Probiotics are live microorganisms that confer health benefits to the host. These beneficial microorganisms are found in different foods and fermented products and are also available as supplements^7^.

Current research has demonstrated alterations in the gut microbiota in DKD, revealing reductions in beneficial strains such as Bifidobacterium and Lactobacillus and an increase in pathogenic bacterial species; these alterations have been associated with elevated levels of inflammatory cytokines, increased oxidative stress, and greater endotoxin translocation factors that contribute to the progression of renal dysfunction^8^.

On the other hand, studies indicate that the reduction of short-chain fatty acids (SCFAs), such as butyrate, acetate, and propionate, worsens the inflammatory state and impairs metabolic homeostasis, intensifying renal damage. In view of this, probiotic intervention emerges as a promising therapeutic strategy for the management of DKD, not only by modulating the gut microbiota and reducing systemic inflammation, but also through its positive effects in suppressing the progression of DKD^7,8,9^. The worsening of DKD leads to the irreversible need for renal replacement therapies.

Considering this scenario, experimental studies in animal models are essential to provide pathophysiological evidence that can underpin clinical research and contribute to the development of effective therapeutic protocols. Thus, this study aimed to evaluate the effects of probiotics on renal function and the redox profile of rats with DKD.

## Methods

Male Wistar rats, eight weeks old, weighing 250–300 g, were allocated to the following groups: Control group (CT, n = 5), which received the vehicles for nicotinamide and streptozotocin, consisting of 0.9% saline and 0.1 M citrate buffer at pH 4.2 on day 1 of the experimental protocol, single dose; type 2 Diabetes Mellitus group (T2DM, n = 5), which received a single dose of nicotinamide (NA; 100 mg/kg) intraperitoneally (i.p.) diluted in 0.9% saline, and after 15 minutes received a single dose of streptozotocin (STZ; 60 mg/kg), i.p., diluted in 0.1 M citrate buffer at pH 4.2 on day 1 of the experimental protocol; and Type 2 Diabetes Mellitus + Probiotic group (T2DM+Prob, n = 5), which were T2DM animals that received probiotic strains (*Bifidobacterium longum*, *Bifidobacterium bifidum*, and *Lactobacillus rhamnosus*; 10^10^ CFU/ mL) by gavage for 6 weeks.

In the sixth week of the experimental protocol, animals from the different groups were placed in metabolic cages for 24-hour urine collection for studies of renal function and oxidative stress. Morphine was administered at a dose of 3 mg/kg, and 30 minutes after, the animals were removed from the metabolic cages and anesthetized with isoflurane (5% for induction and 3% for maintenance). Subsequently, animals underwent laparotomy and terminal blood collection via puncture of the abdominal aorta. The left kidney was removed, conditioned, and stored in a freezer at -80°C for redox studies. All procedures involving animals were carried out in accordance with the ethical standards established by the Animal Use Ethics Committee (CEUA) of the Faculty of Medical Sciences of Santa Casa de São Paulo (approval number 2023/07)^10,11,12^.

### Renal Function Biomarkers

Serum creatinine (CrS) levels were determined by the Jaffé colorimetric method. This method is based on the reaction of creatinine with picric acid in an alkaline medium, forming a yellowish complex whose intensity is proportional to the creatinine concentration in the sample. The reading was performed on a spectrophotometer at 520 nm^13^. The quantification of microalbuminuria was carried out using an enzyme-linked immunosorbent assay (ELISA) for the detection of rat albuminuria (Bethyl Laboratories Inc, Montgomery, USA). This assay was performed on urine samples prepared according to the experimental protocols described by the manufacturer, and the results were expressed in mg/24 h. The glomerular filtration rate (GFR) was estimated by inulin clearance (CIn). After anesthesia, catheterization of the jugular vein was performed with a polyethylene tube (PE 60) for continuous infusion of inulin. In parallel, the carotid artery was catheterized with a polyethylene tube (PE 60) for blood sampling and monitoring of hemodynamic parameters. The concentration of inulin was analyzed according to methodology previously described in the literature by the anthrone method^14^.

### Redox Markers and Antioxidant Expression

Peroxides are found in all body fluids, especially urine. Changes in their levels are considered markers of H_2_O_2_ generation or predictors of the extent of oxidative injury in vivo. Direct measurement of peroxides was performed using the FOX-2 assay. The reading was carried out by spectrophotometry at an absorbance of 560 nm and values expressed as nmol of peroxides per gram of creatinine^15^.

The urinary TBARS assay consisted of adding 0.4 mL of the urine sample to 0.6 mL of distilled water. To this dilution, 1.0 mL of 17.5% TCA and 1.0 mL of thiobarbituric acid (0.6%, pH 2) were added, and all test tubes were kept on ice during this first stage of the process. The solution was homogenized and then placed in a boiling water bath for 20 minutes for reaction with thiobarbituric acid. In the next step, the solution was removed from the water bath, cooled on ice, and 1.0 mL of 70% TCA was added. The solution was homogenized and incubated for 20 minutes in a capped test tube. The solution was then centrifuged for 15 minutes at 3000 revolutions per minute, and the reading was performed by spectrophotometry at an absorbance of 534 nm. The MDA level (nmol) was calculated using the molar extinction coefficient 1.56 × 10^5^ M^-1^ cm^-1^. Values were expressed per gram of creatinine^16^.

Renal tissue glutathione (GSH) was analyzed by the Ellman (DTNB) colorimetric method. Renal tissue was homogenized in 0.1 M phosphate buffer (pH 7.4) containing 1 mM EDTA, followed by centrifugation at 10,000–15,000 × g for 20 minutes at 4°C to obtain the supernatant. To remove proteins, 10% trichloroacetic acid (TCA) was used and the sample was centrifuged again. The treated supernatant was then mixed with phosphate buffer and 1 mM DTNB, reacting with GSH thiols to form a yellow TNB complex, whose absorbance was measured at 412 nm. Quantification was performed based on a 100µM reduced GSH standard curve, and the results were expressed as nmol/mg of protein^17^.

### Western Blotting for Nrf2

The Nuclear Factor Erythroid 2–Related Factor 2 (Nrf2) was assessed in renal tissue samples by western blot. Protein was quantified by the Bradford assay, and 30 µg of protein per lane were separated by electrophoresis on a 10% polyacrylamide gel. Next, proteins were transferred to a nitrocellulose membrane. The membrane was blocked with TBS (50 mM Tris-HCl, pH 7.4, and 150 mM NaCl) containing 0.05% Tween 20 and 5% skim milk (or bovine serum albumin) for one and a half hours at room temperature. The membrane was then incubated overnight at 4°C in a solution containing the primary antibody (Nrf2, Thermo Fisher) and then incubated for 1 hour with an anti-rabbit secondary antibody. Fluorescent immunoblotting was performed and scans were acquired with an infrared imaging system^18^. Statistical analysis: results were expressed as mean ± standard deviation. Variance among groups was analyzed using the one-way ANOVAtest, followed by Tukey’s multiple comparisons post-test in GraphPad Prism version 10 for Windows^®^. Values of p < 0.05 were considered significant. The data used in the analyses are available upon request.

## Results

### Probiotics Improve Renal Function inT2DM Rats

**Figure 1:**
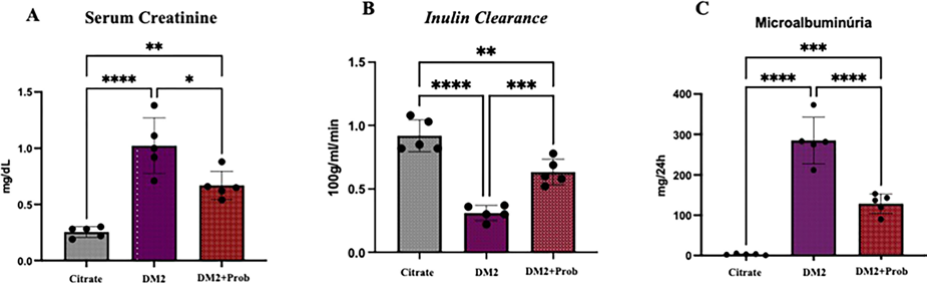
Probiotics improved renal function in T2DM rats. Wistar rats were chemically induced to T2DM and received Probiotics (Prob) by oral gavage for 6 weeks. Citrate: received vehicle; T2DM: Type 2 Diabetes Mellitus; T2DM + Prob: Type 2 Diabetes Mellitus + Probiotics. Group differences were tested by one-way ANOVA, followed by Tukey’s post-test. *p < 0.05 vs. Citrate; **p < 0.05 vs. T2DM; ***p < 0.05 vs. T2DM + Prob.

Renal function was evaluated by serum creatinine levels, microalbuminuria, and inulin clearance. As expected, the T2DM group showed reduced renal function, with elevated serum creatinine and microalbuminuria accompanied by a reduction in GFR (Figures 1A, 1B, and 1C) when compared with the Citrate group. These findings corroborate the establishment of progressive renal damage in diabetic rats, reflecting the loss of glomerular filtration capacity, characteristics of DKD. On the other hand, animals treated with probiotics demonstrated a significant improvement in renal function parameters. Inulin clearance (CIn) was partially restored, suggesting an attenuation of GFR decline (Figure 1C). Likewise, a statistically significant reduction in CrS levels and microalbuminuria was observed, indicating a renoprotective effect of probiotics (Figures 1A and 1B). These findings suggest that modulation of the gut microbiota can positively influence renal function in rats with DKD.

### Probiotics Regulate Redox Signaling in DKD

**Figure 2:**
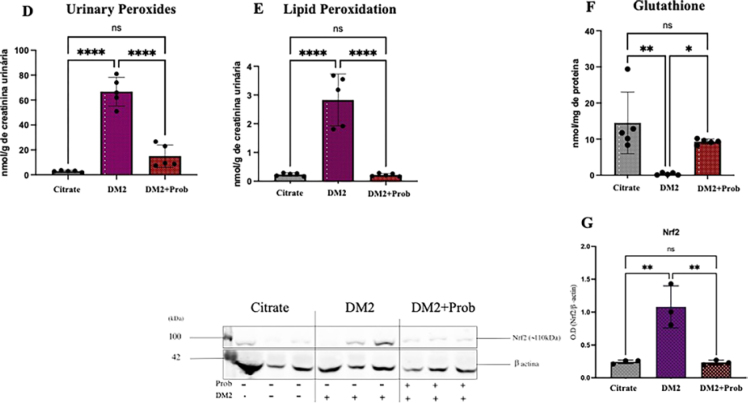
Probiotics regulate redox signaling in DKD. Wistar rats were chemically induced to T2DM and received Probiotics (Prob) by oral gavage for 6 weeks. Citrate: received vehicle; T2DM: Type 2 Diabetes Mellitus; T2DM + Prob: Type 2 Diabetes Mellitus + Probiotic. Group differences were tested by one-way ANOVA, followed by Tukey’s post-test. *p < 0.05 vs. Citrate; **p < 0.05 vs. T2DM; ***p < 0.05 vs. T2DM+Prob.

Lipid and urinary peroxidation were significantly elevated in the T2DM group (Figures 2D and 2E), indicating oxidative stress in diabetic animals. In addition, there was a reduction in GSH concentration and Nrf2 expression in this group (Figures 2F and 2G), evidencing impairment of the antioxidant system. Administration of probiotics in the T2DM + Prob group resulted in a significant reduction in lipid and urinary peroxidation, with lower free radical production and less oxidative damage (Figures 2D and 2E). Moreover, GSH levels and Nrf2 expression showed a significant recovery compared with the T2DM group, indicating an increase in antioxidant defenses via modulation of the gut microbiota and suggesting greater production of short-chain fatty acids, especially butyrate (Figures 2F and 2G).

## Discussion

DKD is a serious disease and an important complication of diabetes. The pathogenesis of DKD is complex and generally influenced by multiple factors. The gut microbiota has been found to play an important role in DKD. In this study, we analyzed renal function and the redox profile of rats with T2DM and DKD. Based on our findings, probiotic administration can beneficially modulate the gut microbiota, positively impacting both renal function and the redox profile of rats with T2DM and DKD^19^.

Our findings demonstrated that probiotics reduced serum creatinine (CrS) and microalbuminuria and increased inulin clearance in T2DM animals. A clinical study in patients with DKD using probiotics showed a significant improvement in renal function biomarkers compared with the placebo group, which corroborates the findings of our study^19^. The improvement observed in renal function parameters in our study may be partially explained by the reduction of uremic toxins derived from bacterial fermentation of amino acids in the gut, a process exacerbated by the sustained hyperglycemia characteristic of DKD. Probiotics have shown a positive effect by reducing the production of these toxins and, consequently, attenuating their deleterious influence on renal function^20^.

The correlation between the gut microbiota and DKD has been widely studied, although the exact mechanisms of this relationship are not yet fully understood. Our findings suggest that short-chain fatty acids (SCFAs), the main fermentation products of the intestinal microbiome – primarily acetate, propionate, and butyrate – may contribute to the improvement of renal function. One study observed that butyrate-producing bacteria are significantly reduced in the gut microbiota of patients with DKD^21^.

Studies have highlighted the interaction between intestinal dysbiosis and the progression of DKD. One study demonstrated that fecal microbiota transplantation can reverse intestinal dysbiosis and, consequently, improve renal function in rats with DKD, suggesting that microbiota modulation is a potential therapeutic resource for renal protection^22,23^. Another clinical study that analyzed the intestinal flora of patients with T2DM and DKD demonstrated that the gut microbiota plays an important role in the pathogenesis of DKD^21^.

Therefore, our findings demonstrate a marked impact on the SCFA elevation pathway, especially the butyrate pathway, which several studies have shown in to be associated with increased antioxidants^21,22,23^.

The findings of this work demonstrated that redox metabolites were modulated when T2DM animals were treated with probiotics, with significant expression of NRF2, an important transcription factor in the antioxidant cascade that prevents redox disturbances and, consequently, elevation of the inflammatory profile, advanced glycation end products (AGEs), and progression of DKD^24,25^. One study demonstrated that butyrate restored renal function and attenuated inflammation, apoptosis, and renal fibrosis in a preclinical model^24^.

We hypothesize that probiotic supplementation modulated Nrf2 redox signaling through specific SCFA receptors, inducing nuclear translocation of Nrf2^25^. This was demonstrated in our study through the nuclear accumulation of Nrf2 in T2DM animals, as revealed by the increased levels this parameter. Probiotic supplementation, however, reduced this parameter, indicating a decrease in redox disturbances in these animals. These findings open new perspectives for the use of probiotics as therapeutic adjuvants in the prevention of T2DM complications and the progression of DKD.

## Conclusion

Probiotics demonstrated potential as a therapeutic nutritional agent in renal protection. Moreover, their impact on the redox profile suggests that their supplementation may represent a complementary strategy to attenuate the progression of DKD, one of the most severe complications of T2DM. Thus, the inclusion of probiotics in the diet, together with lifestyle changes, may help reduce the need for renal replacement therapy, promoting an effective preventive and therapeutic approach.

## Data Availability

The datasets generated and/or analyzed during the current study are not publicly available but are available from the corresponding author on reasonable request.
