# Supplementary material for: The role of probiotics in modulating the gut microbiota as a potential inhibitor of diabetic kidney disease progression
Source: J Bras Nefrol. 2026 Jan 12;48(2):e20250143. doi: 10.1590/2175-8239-JBN-2025-0143en (PMC12797498; doi:10.1590/2175-8239-JBN-2025-0143en)
Supplement: Translation

# Translation: O papel dos probióticos na modulação da microbiota intestinal como potencial inibidor da progressão da doença renal diabética

**DOI:** 10.1590/2175-8239-JBN-2025-0143pt

**Authors:** Vitoria Cecilia Souza Costa, Monique Moreira Pinheiro, Giulia Triolo Cabreira, Isabella Bacci Bustelli, Julia Ferreira Santos, Sara Ventura, Luciana Soares Costa Santos, Maria de Fatima Fernandes Vattimo, Eloiza de Oliveira Silva

**Affiliations:** 1 Universidade de São Paulo, Escola de Enfermagem, São Paulo, SP, Brasil.; 2 Universidade de São Paulo, Faculdade de Medicina, Instituto do Coração, Laboratório de Biologia Vascular, São Paulo, SP, Brasil.; 3 Faculdade de Ciências Médicas da Santa Casa de São Paulo, Departamento de Enfermagem e Fisiologia, São Paulo, SP, Brasil.

**Keywords:** Doença renal diabética, Diabetes mellitus tipo 2, Probióticos, Estresse oxidativo

## Abstract

**Introdução:**

A disbiose intestinal é comumente observada em pacientes com doença renal diabética e pode contribuir para sua patogênese. Entre os metabólitos microbianos, o butirato desempenha papel fundamental na regulação de proteínas antioxidantes no diabetes mellitus tipo 2. Com base nisso, levantamos a hipótese de que a administração de probióticos em ratos diabéticos poderia modular o perfil redox, atenuando, assim, a progressão da doença renal.

**Métodos:**

Estudo *in vivo* utilizando ratos *Wistar* machos (8 semanas, 250–300 g; n = 15), randomizados em três grupos (n = 5/grupo): Controle (veículos: solução salina 0,9% e citrato 0,1 M; pH 4,2, i.p., no dia 1); DM2 (nicotinamida 100 mg/kg, i.p., seguida de estreptozotocina 60 mg/kg, i.p., em tampão citrato 0,1 M, pH 4,2); e DRD + Prob (protocolo D + probiótico multicepas *Bifidobacterium longum, Bifidobacterium bifidum e Lactobacillus rhamnosus*, 10^10^ UFC/mL via gavagem por 6 semanas). Parâmetros avaliados: creatinina sérica, *clearance de* inulina, microalbuminúria, peróxidos urinários e lipídicos, glutationa e Fator 2 Relacionado ao Eritroide Nuclear 2.

**Resultados:**

O tratamento probiótico aumentou significativamente a expressão de Nrf2 e os níveis de glutationa. Por outro lado, reduziu a peroxidação urinária e lipídica. Além de atenuar o estresse oxidativo, os probióticos melhoraram a função renal, com redução da creatinina sérica e da microalbuminúria e aumento do *clearance* de inulina.

**Conclusão:**

Os achados indicam que os probióticos preveniram a progressão da DRD, provavelmente modulando o estresse oxidativo por meio da microbiota intestinal. Esses resultados sugerem que os probióticos podem atuar como agentes renoprotetores, reduzindo potencialmente a morbidade da DRD no DM2.

## Introdução

O diabetes mellitus tipo 2 (DM2) é definido como um conjunto de alterações metabólicas caracterizado por níveis elevados de glicemia sustentada, decorrentes da diminuição da produção de insulina pancreática ou da redução da sensibilidade à insulina. É responsável por 91% da carga global de diabetes (DM) e constitui a causa mais comum de desenvolvimento de doença renal crônica (DRC) e doença renal crônica estágio terminal (DRCT). O DM2 está associado a fatores de risco como doença cardiovascular (DCV), incluindo hipertensão, dislipidemia, obesidade, resistência à insulina e intolerância à glicose^1,2^.

Apesar de as complicações do DM2 apresentarem origem multifatorial, a hiperglicemia sustentada — que contribui para o aumento de citocinas inflamatórias e do estresse oxidativo, levando a complicações a longo prazo — demonstra ser a principal causa de lesão tecidual no rim diabético. Essa condição gera complicações crônicas importantes, como retinopatia, neuropatia, doença cardiovascular e doença renal diabética (DRD)^3,4^.

A DRD, definida clinicamente pela presença de albuminúria ≥30 mg/g e/ou taxa de filtração glomerular < 60 mL/min/1,73 m^2^, persistente por pelo menos 3 meses, independentemente da etiologia, é uma complicação crônica que resulta em danos progressivos e comprometimento da função renal^5,6^.

A DRD é uma preocupação significativa em todo o mundo, afetando milhões de pessoas com DM2. Aproximadamente 50% dos pacientes com DRD apresentam microalbuminúria que progride para macroalbuminúria, uma das complicações microvasculares mais importantes da doença. Além disso, cerca de metade de todos os casos de DRCT está relacionada a complicações do DM^5^.

Sendo assim, a DRD é uma das principais causas de início de terapia de substituição renal e está associada ao aumento da morbidade e mortalidade. Diante disso, torna-se urgente explorar tratamentos eficazes e de baixo custo que possam mitigar esses desfechos na vigência de DRD^3,4^.

Além do tratamento tradicional, como o controle glicêmico e a terapia medicamentosa, novas abordagens terapêuticas estão sendo exploradas. Entre elas, destaca-se o papel promissor dos probióticos na gestão da DRD. Estudos recentes têm demonstrado que os probióticos podem desempenhar um papel importante na prevenção e no tratamento da DRD, por meio de diferentes mecanismos, como a modulação da microbiota intestinal^7,8^.

Os probióticos são microrganismos ativos que conferem benefícios à saúde do hospedeiro. Esses microrganismos benéficos são encontrados em diferentes alimentos e produtos fermentados, além de estarem disponíveis na forma de suplementos^7^.

Pesquisas atuais têm demonstrado alterações da microbiota intestinal na DRD, revelando reduções de cepas benéficas, como *Bifidobacterium* e *Lactobacillus*, e aumento de espécies bacterianas patogênicas. Essas alterações foram associadas a níveis elevados de citocinas inflamatórias, aumento do estresse oxidativo e maior translocação de endotoxinas, fatores que contribuem para a progressão da disfunção renal^8^.

Por outro lado, estudos indicam que a redução dos ácidos graxos de cadeia curta (AGCC), como butirato, acetato e propionato, agrava o quadro inflamatório e prejudica a homeostase metabólica, intensificando os danos renais. Diante disso, a intervenção com probióticos surge como uma estratégia terapêutica promissora para o manejo da DRD, não apenas modulando a microbiota intestinal e reduzindo a inflamação sistêmica, mas também demonstrando efeitos positivos na supressão da progressão da doença^7,8,9^. O agravamento da DRD leva à necessidade irreversível de terapias de substituição renal.

Considerando esse cenário, estudos experimentais em modelos animais são essenciais para fornecer evidências fisiopatológicas que possam fundamentar pesquisas clínicas e contribuir para a elaboração de protocolos terapêuticos eficazes. Dessa forma, este estudo teve como objetivo avaliar os efeitos dos probióticos na função renal e no perfil redox de ratos com DRD.

## Métodos

Ratos *Wistar* machos, com oito semanas de idade e pesando entre 250-300 g, foram distribuídos nos seguintes grupos: **Controle (CT; n = 5)**: animais que receberam os veículos da nicotinamida e da estreptozotocina, consistindo em solução salina 0,9% e tampão citrato 0,1 M (pH 4,2), no 1º dia do protocolo experimental, em dose única; **Diabetes mellitus tipo 2 (DM2; n = 5)**: animais que receberam nicotinamida (NA; 100 mg/kg), intraperitoneal (i.p.), em dose única, diluída em solução salina 0,9%. Em seguida, após 15 minutos, receberam estreptozotocina (STZ; 60 mg/kg), i.p., em dose única, diluída em tampão citrato 0,1 M (pH 4,2), no 1º dia de protocolo experimental; **Diabetes mellitus tipo 2 + Probiótico (DM2 + Prob; n = 5)**: animais DM2 que receberam probióticos contendo as cepas *Bifidabacterium longum*, *Bifidabacterium bifidum* e *Lactobacillus rhamnosus* (10^10^ UFC/mL), administrados por via oral, por gavagem, durante 6 semanas.

Na sexta semana do protocolo experimental, os animais dos diversos grupos foram colocados em gaiolas metabólicas para coleta de urina de 24 horas. As amostras urinárias foram utilizadas para a realização de estudos de função renal e estresse oxidativo. Após a retirada das gaiolas metabólicas, os animais foram anestesiados com isoflurano (5% indução e 3% manutenção). Trinta minutos antes da indução anestésica, foi administrada morfina na dose de 3 mg/kg. Em seguida, os animais passaram pelos procedimentos necessários para os estudos de função renal pela técnica de determinação do *clearance* de inulina. Posteriormente, foram submetidos à laparotomia e à coleta de sangue terminal por meio da punção da aorta abdominal. O rim esquerdo foi removido, acondicionado e armazenado em refrigerador a -80ºC para estudos redox. Todos os procedimentos envolvendo animais foram realizados de acordo com os padrões éticos estabelecidos pela Comissão de Ética no Uso de Animais (CEUA) da Faculdade de Ciências Médicas da Santa Casa de São Paulo (sob o número de aprovação 2023/07)^10,11,12^.

### Biomarcadores de Função Renal

Os níveis séricos de creatinina (CrS) foram determinados pelo método colorimétrico conhecido como método de Jaffé. Esse método baseia-se na reação da creatinina com o ácido pícrico em meio alcalino, formando um complexo de cor amarelada cuja intensidade é proporcional à concentração de creatinina na amostra. A leitura foi realizada em espectrofotômetro a 5*2*0 nm^13^. A quantificação da microalbuminúria foi realizada por meio de ensaio de imunoabsorção enzimática (ELISA) para detecção de albuminúria de rato (*Bethyl Laboratories Inc*., Montgomery, EUA). Esse ensaio foi realizado em amostras urinárias preparadas de acordo com os protocolos experimentais descritos pelo fabricante, e os resultados foram expressos em mg/24h. A taxa de filtração glomerular (TFG) foi estimada por meio do *clearance* de inulina (Clin). Para esse procedimento, os animais foram previamente anestesiados com isoflurano inalatório (5% indução e 3% manutenção). Trinta minutos antes da indução anestésica, foi administrada morfina na dose de 3 mg/kg. Em seguida, realizou-se cateterização da veia jugular com um tubo de polietileno (PE 60) para infusão contínua de inulina. Paralelamente, a artéria carótida foi cateterizada com um tubo de polietileno (PE 60) para coleta de amostras sanguíneas e monitoramento dos parâmetros hemodinâmicos. A concentração de inulina foi analisada conforme metodologia previamente descrita na literatura, utilizando o método de antrona^14^.

### Marcadores Redox e Expressão Antioxidante

Os peróxidos são encontrados em todos os fluidos corporais, especialmente na urina. Alterações em seus níveis são consideradas marcadores de geração de H_2_O_2_ ou preditores da extensão da lesão oxidativa *in vivo*. A mensuração direta de peróxidos foi realizada por meio do método de análise FOX- 2. A leitura foi realizada por espectrofotometria, em absorbância de 560 nm. Os valores foram estabilizados por grama de creatinina urinária e expressos em nmol de peróxidos/grama de creatinina^15^.

A dosagem de TBARS na urina consistiu na adição de 0,4 mL da amostra de urina a 0,6 mL de água destilada. Foram acrescentados, nessa diluição, 1,0 mL de TCA a 17,5% e 1,0 mL de ácido tiobarbitúrico (0,6%, pH 2), sendo que todos os tubos de ensaio contendo a solução foram mantidos em gelo durante essa primeira etapa do processo. A solução foi homogeneizada e, em seguida, colocada em água fervente (banho-maria) durante 20 minutos para reação com o ácido tiobarbitúrico. Na etapa seguinte, a solução foi retirada do banho-maria, resfriada em gelo e adicionou-se 1,0 mL de TCA a 70%. A solução foi homogeneizada e incubada por 20 minutos em tubo de ensaio tampado. Ao final, foi centrifugada por 15 minutos a 3000 rotações por minuto, e a leitura foi realizada por espectrofotometria, em absorbância de 534 nm. A quantidade de MDA, expressa em nmol, foi calculada usando o coeficiente de extinção molar de 1,56 × 10^5^ M^-1^ cm^-1^. Os valores foram expressos por grama de creatinina^16^.

A análise de glutationa (GSH) no tecido renal foi realizada pelo método colorimétrico de *Ellman* (DTNB). O tecido renal foi homogeneizado em tampão fosfato 0,1 M (pH 7,4) contendo EDTA 1 mM, seguido de centrifugação a 10.000–15.000 × g por 20 minutos, a 4°C, para obtenção do sobrenadante. Para remover proteínas, utilizouse ácido tricloroacético (TCA 10%) e realizou-se nova centrifugação. O sobrenadante tratado foi então misturado com tampão fosfato e DTNB 1 mM, reagindo com os tióis da GSH e formando o complexo amarelo TNB, cuja absorbância foi medida a 412 nm. A quantificação foi realizada com base em uma curva padrão de GSH 100 µM reduzida, e os resultados foram expressos em nmol/mg de proteína^17^.

### *Western Blotting* Para Nrf2

O Fator 2 Relacionado ao Eritroide Nuclear 2 (Nrf2) foi avaliado em amostras de tecido renal por *western blot*. A proteína foi quantificada pelo ensaio de *Bradford*, e 30 µg de proteína por canaleta foram separados por eletroforese em gel de poliacrilamida a 10%. Após a separação, as proteínas foram transferidas para uma membrana de nitrocelulose. Essa membrana foi bloqueada com TBS (Tris-HCl 50 mM, pH 7,4, e NaCl 150 mM) contendo 0,05% de *Tween* 20 e leite desnatado a 5% (ou albumina bovina), por uma hora e meia, à temperatura ambiente. Em seguida, foi incubada durante a noite, a 4º C, em solução contendo o anticorpo primário (Nrf2, *ThermoFisher*). Após esse período, a membrana foi submetida à incubação por 1 hora com o anticorpo secundário anti-coelho. Foi realizada imunotransferência fluorescente e posterior digitalização com o sistema de imagem por infravermelho^18^. Para a análise estatística, os resultados foram expressos como média ± desvio padrão. A variância entre os grupos foi analisada por meio do teste *One Way* ANOVA, seguido do pósteste de comparações múltiplas de *Tukey*, utilizando o programa estatístico *GraphPad Prism version 10* (Windows^®^). Foram considerados significativos os valores de p < 0,05. Os dados utilizados nas análises estão disponíveis mediante solicitação.

## Resultados

### Probióticos Melhoram a Função Renal de ratos DM2

A função renal foi avaliada pelos níveis de creatinina sérica, microalbuminúria e *clearance* de inulina. Como esperado, o grupo DM2 apresentou função renal reduzida, com níveis elevados de creatinina sérica e microalbuminúria, acompanhados por redução da TFG (Figuras 1A, 1B e 1C) quando comparado ao grupo Citrato. Esses achados corroboram a instalação de dano renal progressivo nos ratos diabéticos, refletindo a perda da capacidade de filtração glomerular, característica da DRD. Por outro lado, os animais tratados com probióticos demonstraram melhora significativa nos parâmetros de função renal. O Clin foi parcialmente restaurado, sugerindo atenuação do declínio da TFG (Figura 1B). Da mesma forma, observou-se uma redução estatisticamente significativa dos níveis de CrS e microalbuminúria, indicando um efeito renoprotetor dos probióticos (Figuras 1A e 1C). Esses achados sugerem que a modulação da microbiota intestinal pode influenciar positivamente a função renal de ratos com DRD.

**Figura 1.** Probióticos melhoram a função renal de ratos DM2. Ratos Wistar foram induzidos ao DM2 quimicamente ou receberam Probióticos (Prob) por via oral gavagem, por 6 semanas. Citrato: receberam veículo; DM2: Diabetes Mellitus tipo 2; DM2 + Prob: Diabetes Mellitus tipo 2 + Probiótico. As diferenças estatísticas foram testadas por ANOVA unidirecional, seguida de pós-teste de Tukey. *p < 0,05 vs. Citrato; **p < 0,05 vs. DM2, ***p < 0,05 vs. DM2 + Prob.

### Probióticos Regulam a Sinalização Redox na DRD

A peroxidação lipídica e urinária esteve significativamente elevada no grupo DM2 (Figuras 2D e 2E), indicando estresse oxidativo nos animais diabéticos. Além disso, houve redução na concentração de GSH e na expressão de Nrf2 nesse grupo (Figuras 2F e 2G), evidenciando comprometimento do sistema antioxidante. A administração de probióticos no grupo DM2 + Prob resultou em redução significativa da peroxidação lipídica e urinária, demonstrando menor produção de radicais livres e menor dano oxidativo (Figuras 2D e 2E). Além disso, os níveis de GSH e a expressão de Nrf2 apresentaram recuperação significativa em relação ao grupo DM2, indicando elevação das defesas antioxidantes via modulação da microbiota intestinal, sugerindo maior produção de ácidos graxos de cadeia curta, principalmente butirato (Figuras 2F e 2G).

**Figura 2.** Probióticos regulam a sinalização redox na DRD. Ratos *Wistar* foram induzidos ao DM2 quimicamente ou receberam Probióticos (Prob) por via oral gavagem, por 6 semanas. Citrato: receberam veículo; DM2: Diabetes Mellitus tipo 2; DM2 + Prob: Diabetes Mellitus tipo 2 + Probiótico. As diferenças estatísticas foram testadas por ANOVA unidirecional, seguida de pós-teste de *Tukey*. *p < 0,05 vs. Citrato; **p < 0,05 vs. DM2; ***p < 0,05 vs. DM2 + Prob.

## Discussão

A DRD é uma doença grave e uma complicação importante do diabetes. Sua patogênese é complexa e geralmente influenciada por múltiplos fatores. Descobriu-se que a microbiota intestinal desempenha um papel importante na DRD. Neste estudo, analisamos a função renal e o perfil redox de ratos com DM2 e DRD. Com base em nossos achados, sugerimos que a administração de probióticos pode modular a microbiota intestinal de maneirabenéfica, impactando positivamente tanto a função renal quanto o perfil redox de ratos com DM2 e DRD^19^.

Nossas descobertas demonstraram que os probióticos reduziram a CrS e a microalbuminúria, além de aumentarem o *clearance* de inulina nos animais DM2. Além disso, um estudo clínico envolvendo pacientes com DRD em uso de probióticos mostrou uma melhora significativa nos biomarcadores de função renal em comparação ao grupo placebo, o que corrobora os achados do nosso estudo^19^. A melhora observada nos parâmetros de função renal pode ser parcialmente explicada pela redução das toxinas urêmicas derivadas da fermentação bacteriana de aminoácidos no intestino, um processo exacerbado pela hiperglicemia sustentada característica da DRD. Os probióticos demonstraram impacto positivo ao reduzir a produção dessas toxinas e, consequentemente, atenuar sua influência deletéria sobre a função renal^20^.

A correlação entre a microbiota intestinal e a DRD vem sendo amplamente estudada, embora os mecanismos exatos dessa relação ainda não estejam totalmente esclarecidos. Nossas descobertas sugerem que os ácidos graxos de cadeia curta (AGCC), principais produtos da fermentação do microbioma intestinal — especialmente acetato, propionato e butirato — podem contribuir para a melhora da função renal. Um estudo observou que bactérias produtoras de butirato estão significativamente reduzidas na microbiota intestinal de pacientes com DRD^21^.

Estudos têm destacado a interação entre disbiose intestinal e a progressão da DRD. Uma pesquisa demonstrou que o transplante de microbiota fecal pode reverter a disbiose intestinal e, consequentemente, melhorar a função renal em ratos com DRD, sugerindo que a modulação da microbiota é um potencial recurso terapêutico para a proteção renal^22,23^. Outro estudo clínico que analisou a flora intestinal de pacientes com DM2 e DRD demostrou que a microbiota intestinal desempenha um papel importante na patogênese da DRD^21^.

Portanto, sugerimos que nossos achados tenham evidenciado um impacto expressivo na via de elevação dos AGCC, principalmente na via do butirato, que demostrou em diversos estudos estar relacionada ao aumento de antioxidantes^21,22,23^.

Os achados deste trabalho demonstraram que os metabólitos redox foram modulados quando animais DM2 foram tratados com probióticos, com expressão significativa de Nrf2, um fator de transcrição importante na cadeia antioxidante, que impede distúrbios redox e, consequentemente, a elevação do perfil inflamatório, dos produtos finais de glicação avançada (AGES) e a progressão da DRD^24,25^. Um estudo demonstrou que o butirato restaurou a função renal e atenuou a inflamação, a apoptose e a fibrose renal em um modelo pré-clínico^24^.

Sugerimos que a suplementação com probióticos modulou a sinalização redox de Nrf2 por meio de receptores específicos de AGCC, induzindo a translocação nuclear de Nrf2^25^. Isso foi demonstrado em nosso estudo pelo acúmulo nuclear de Nrf2 nos animais DM2, evidenciado pela elevação desse marcador. Por outro lado, a suplementação com probióticos reduziu esse parâmetro, demonstrando diminuição dos distúrbios redox nesses animais. Esses achados abrem novas perspectivas para o uso de probióticos como adjuvantes terapêuticos na prevenção de complicações da DM2 e na progressão da DRD.

## Conclusão

Os probióticos demonstraram ser um potencial recurso terapêutico como agentes nutricionais na proteção renal. Além disso, o impacto desses microrganismos no perfil redox sugere que sua suplementação pode representar uma estratégia complementar para atenuar a progressão da DRD, um dos mais graves complicadores do DM2. Dessa forma, a inclusão de probióticos na alimentação, aliada a mudanças no estilo de vida, pode contribuir para reduzir a necessidade de terapia de substituição renal, promovendo uma abordagem preventiva e terapêutica eficaz.

## Data Availability

Os conjuntos de dados gerados e/ou analisados durante o presente estudo estão disponíveis mediante solicitação razoável ao autor correspondente.

## Financiamento

Este estudo foi financiado pela Fundação de Amparo à Pesquisa da Faculdade de Ciências Médicas da Santa Casa de São Paulo.
